# Supplementary material for: Urinary Metabolite Profiles of Participants with Overweight and Obesity Prescribed a Weight Loss High Fruit and Vegetable Diet: A Single Arm Intervention Study
Source: Nutrients. 2024 Dec 17;16(24):4358. doi: 10.3390/nu16244358 (PMC11677377; doi:10.3390/nu16244358)
Supplement: Supplementary file 1 [file nutrients-16-04358-s001.zip › nutrients-3338096-supplementary.pdf]

Supplementary Table S1: Univariate Analysis Result for each urinary metabolites comparing at each time point

	Baseline (µM)	Week 2 (µM)	Week 10 (µM)	Difference Baseline and Week 2		Difference Baseline and Week 10		Difference Week 2 and Week 10	
**Name**	**Mean (SD)**	**Mean (SD)**	**Mean (SD)**	**p-value**	**p.value.origin**	**p-value**	**p.value.origin**	**p-value**	**p.value.origin**
HMDB0000034 **(Adenine)**	99.04 (113.88)	110.39 (112.36)	119.88 (94.34)	0.85 (W)	0.85	0.21 (W)	0.16 (W)	0.13(W)	0.13
HMDB0000042 ŧ **(Acetic acid)**	76.89 (58.05)	130.61 (137.34)	106.23 (66.86)	**0.001 (W)**	**0.001**	**0.08 (W)**	**<0.05 (W)**	0.12(W)	0.12
HMDB0000045 **(Adenosine monophosphate)**	13.72 (15.08)	21.80 (26.53)	16.72 (11.78)	**0.02 (W)**	**0.02**	0.22 (W)	0.26 (W)	0.09	0.09
HMDB0000050 **(Adenosine)**	29.04 (33.20)	31.88 (30.03)	35.48 (28.99)	0.15 (W)	0.15	0.13 (W)	0.21 (W)	0.87	0.87
HMDB0000060 **(Acetoacetic acid)**	139.82 (228.75)	140.67 (167.68)	139.93 (139.88)	0.71	0.71	0.47 (W)	0.63	0.87	0.87
HMDB0000062 **(L-Carnitine)**	70.06 (90.54)	94.68 (115.42)	83.66 (87.34)	0.33	0.33	0.23 (W)	0.21	0.87	0.87
HMDB0000064 **(Creatine)**	271.51 (265.57)	353.20 (408.86)	900.71 (2645.73)	0.63	0.63	0.07(W)	0.09 (W)	0.18 (W)	0.18
HMDB0000072 **(cis-Aconitic acid)**	250.74 (229.55)	338.62 (337.79)	370.20 (644.11)	**0.03 (W)**	**0.03**	0.41 (W)	0.61 (W)	0.11 (W)	0.11
HMDB0000087 ŧ **(Dimethylamine)**	60.85 (154.02)	199.47 (273.16)	282.46 (274.65)	**0.004 (W)**	**0.004**	**0.0002 (W)**	**< 0.0001 (W)**	**0.04 (W)**	**0.04**
HMDB0000092 **(Dimethylglycine)**	24.65 (26.46)	30.06 (25.87)	30.16 (29.11)	0.51 (W)	0.51	0.47 (W)	0.78 (W)	0.57 (W)	0.57
HMDB0000094 **(Citric acid)**	2261.06 (2183.85)	3870.34 (5201.32)	2998.83 (2443.41)	**0.0005**	**0.0005**	0.15 (W)	0.14 (W)	0.12 (W)	0.12
HMDB0000097 ŧ **(Choline)**	28.61 (34.12)	20.20 (22.06)	33.32 (76.41)	**0.03 (W)**	**0.03**	**0.74 (W)**	**0.04 (W)**	0.94 (W)	0.94
HMDB0000108 **(Ethanol)**	665.83 (1618.35)	423.78 (679.13)	274.04 (351.61)	0.85 (W)	0.85	0.87 (W)	0.54	0.81 (W)	0.81
HMDB0000112 **(gamma-Aminobutyric acid)**	101.03 (97.0)	128.35 (138.14)	89.73 (72.43)	**0.04**	**0.04**	0.97 (W)	0.35 (W)	**0.001 (W)**	**0.001**
HMDB0000119 **(Glyoxylic acid)**	261.87 (227.42)	371.54 (327.43)	306.11 (213.13)	**0.004 (W)**	**0.004**	0.41 (W)	1.00 (W)	**0.003 (W)**	**0.002**

HMDB0000122 **(D-Glucose)**	225.16 (222.39)	325.54 (541.69)	194.74 (125.39)	**<0.05 (W)**	**<0.05**	0.86 (W)	0.22	**0.001 (W)**	**0.001**
**HMDB0000122.1**	794.29 (891.23)	991.91 (1168.5)	948.03 (792.50)	**0.04**	**0.04**	0.21 (W)	0.08	0.74	0.74
HMDB0000123 **(Glycine)**	256.33 (296.13)	331.28 (385.51)	291.28 (417.64)	0.83 (W)	0.83	0.79 (W)	0.31	0.41 (W)	0.41
HMDB0000127 **(D-Glucuronic acid)**	114.97 (92.65)	171.22 (400.34)	71.84 (54.13)	0.10 (W)	0.10	**0.05 (W)**	**< 0.0001 (W)**	**0.0004 (W)**	**0.0004**
HMDB0000128 **(Guanidoacetic acid)**	117.81 (110.03)	158.43 (211.69)	129.07 (102.61)	0.47 (W)	0.47	0.62 (W)	0.93 (W)	0.57 (W)	0.57
HMDB0000131 **(Glycerol)**	27.09 (50.24)	7.20 (8.85)	4.21 (3.22)	0.61 (W)	0.61	**0.03 (W)**	**0.03 (W)**	**0.01**	**0.01**
HMDB0000133 **(Guanosine)**	47.02 (95.26)	44.78 (63.09)	92.76 (279.46)	0.11	0.11	**0.02 (W)**	**0.008 (W)**	0.21 (W)	0.21
HMDB0000134 ǂ **(Fumaric acid)**	171.13 (176.57)	279.58 (215.40)	283.48 (220.37)	**0.002 (W)**	**0.002**	**0.01 (W)**	**0.02 (W)**	0.24	0.24
HMDB0000142 **(Formic acid)**	182.77 (169.84)	149.00 (176.86)	161.20 (144.20)	0.42 (W)	0.42	0.53 (W)	0.88 (W)	0.35 (W)	0.35
HMDB0000143 **(D-Galactose)**	464.55 (553.24)	600.90 (638.65)	601.26 (527.85)	**0.05 (W)**	**<0.05**	0.08 (W)	0.11 (W)	0.76 (W)	0.76
HMDB0000148 ǂ **(Glutamic acid)**	256.90 (312.19)	178.90 (201.65)	156.61 (127.75)	**0.01 (W)**	**0.01**	**0.36 (W)**	**0.024 (W)**	0.75 (W)	0.75
HMDB0000149 **(Ethanolamine)**	378.06 (405.74)	429.33 (403.82)	383.70 (294.66)	0.91 (W)	0.91	0.70 (W)	0.61 (W)	0.81 (W)	0.81
HMDB0000156 **(Malic acid)**	63.32 (70.30)	78.78 (125.87)	52.38 (46.77)	0.75	0.75	0.93 (W)	0.27	0.12	0.12
HMDB0000157 **(Hypoxanthine)**	36.55 (46.75)	49.14 (68.32)	40.94 (28.76)	0.35 (W)	0.35	0.10 (W)	0.43 (W)	0.95 (W)	0.95
HMDB0000158 ǂ **(L-Tyrosine)**	314.26 (309.82)	533.01 (651.49)	471.12 (490.67)	**0.03 (W)**	**0.03**	**0.13 (W)**	**<0.05 (W)**	0.82	0.82
HMDB0000159 **(Phenylalanine)**	170.69 (296.26)	81.39 (100.12)	64.63 (58.41)	0.24 (W)	0.24	**0.20 (W)**	**0.02 (W)**	0.08	0.08
HMDB0000161 **(L-Alanine)**	35.69 (34.23)	44.31 (51.33)	35.88 (28.46)	0.76	0.76	0.66 (W)	0.47	0.35	0.35
HMDB0000162 **(Proline)**	68.97 (49.36)	108.64 (97.74)	94.69 (63.92)	**0.02 (W)**	**0.02**	0.10 (W)	0.32 (W)	0.15 (W)	0.15
HMDB0000164 **(Methylamine)**	101.87 (107.85)	151.62 (211.96)	115.35 (83.23)	**0.003**	**0.003**	0.24 (W)	0.07	**0.04**	**0.04**

HMDB0000167 (**L-Threonine)**	254.83 (391.60)	303.89 (458.24)	216.76 (171.88)	0.13	0.13	0.33 (W)	0.61	0.26	0.26
HMDB0000168 (**L-Asparagine)**	48.50 (59.84)	53.27 (106.62)	22.20 (30.81)	0.75 (W)	0.75	**0.02 (W)**	**0.003**	**0.04 (W)**	**0.04**
HMDB0000172 **(Isoleucine)**	16.03 (20.01)	17.15 (15.92)	15.80 (10.46)	0.12 (W)	0.12	0.30 (W)	0.64	0.07 (W)	0.07
HMDB0000174 (**L-Fucose)**	40.94 (48.87)	52.47 (61.84)	48.00 (47.8)	0.51 (W)	0.51	0.67 (W)	0.99 (W)	0.50 (W)	0.50
HMDB0000177 ŧ **(Histidine)**	1308.04 (1363.49)	2215.59 (2685.40)	2154.74 (2133.23)	**0.05 (W)**	**<0.05**	**0.14 (W)**	**0.03 (W)**	0.89 (W)	0.89
HMDB0000182 **(Lysine)**	57.85 (68.96)	87.32 (94.62)	80.37 (66.93)	**0.03 (W)**	**0.03**	0.07 (W)	0.09 (W)	0.62 (W)	0.62
HMDB0000187 **(Serine)**	202.53 (253.16)	311.84 (501.69)	198.62 (214.74)	0.81 (W)	0.81	0.95 (W)	0.91 (W)	0.48 (W)	0.48
HMDB0000190 **(Lactic acid)**	145.75 (267.11)	86.02 (133.28)	95.18 (112.49)	0.12 (W)	0.12	0.57 (W)	0.11 (W)	0.70	0.70
HMDB0000191 (**L-Aspartic acid)**	277.97 (375.82)	357.01 (562.08)	284.86 (221.38)	0.58	0.58	0.35 (W)	0.91	0.65	0.65
HMDB0000192 (**L-Cystine)**	31.98 (30.93)	42.69 (49.83)	33.88 (36.77)	0.06	0.06	0.84 (W)	0.70	**0.03**	**0.03**
HMDB0000192.1	119.15 (144.29)	151.52 (210.74)	113.76 (86.33)	0.42 (W)	0.42	0.63 (W)	0.94	0.38 (W)	0.37
HMDB0000194 **(Anserine)**	77.95 (176.36)	56.95 (84.82)	32.40 (33.12)	0.77 (W)	0.77	0.22 (W)	0.07 (W)	**0.02**	**0.02**
HMDB0000195 **(Inosine)**	5.47 (5.72)	4.72 (5.76)	6.80 (12.34)	0.31 (W)	0.31	0.93 (W)	0.27 (W)	0.97 (W)	0.97
HMDB0000201 **(L-Acetylcarnitine)**	29.44 (34.44)	53.96 (83.85)	33.68 (40.16)	**<0.05 (W)**	**<0.05**	0.50 (W)	0.99 (W)	0.15	0.15
HMDB0000202 **(Methylmalonic acid)**	52.66 (73.39)	39.98 (42.73)	35.28 (43.81)	0.63	0.63	0.63 (W)	0.10	0.15	0.15
HMDB0000202.1	90.34 (108.63)	76.15 (91.07)	73.21 (69.03)	0.40 (W)	0.40	0.82 (W)	0.36 (W)	0.88 (W)	0.88
HMDB0000208 (**Oxoglutaric acid)**	108.46 (122.93)	142.06 (356.23)	96.49 (88.31)	0.90 (W)	0.90	0.99 (W)	0.24	0.29 (W)	0.29
HMDB0000209 **(Phenylacetic acid)**	2.20 (2.32)	3.28 (3.46)	2.26 (1.96)	**0.03 (W)**	**0.03**	0.49 (W)	0.98 (W)	**0.03 (W)**	**0.03**
HMDB0000211 **(myo-Inositol)**	101.62 (95.55)	159.20 (188.12)	106.42 (96.13)	**0.05 (W)**	**<0.05**	0.76 (W)	0.93 (W)	**0.03 (W)**	**0.03**

HMDB0000237 (**Propionic acid)**	11.83 (14.02)	10.20 (11.76)	10.90 (9.73)	0.08	0.08	0.98 (W)	0.14	0.76	0.76
HMDB0000239 (**Pyridoxine)**	13.90 (17.57)	7.24 (5.36)	8.91 (7.02)	0.42 (W)	0.42	0.50 (W)	0.46 (W)	0.97	0.97
HMDB0000243 (**Pyruvic acid)**	31.68 (40.53)	18.84 (22.40)	16.66 (17.96)	0.05	0.05	**0.18 (W)**	**0.002 (W)**	**0.03 (W)**	**0.03**
HMDB0000243.1	10.54 (12.96)	10.42 (14.00)	5.73 (5.31)	0.80	0.80	**0.20 (W)**	**0.04 (W)**	**0.04 (W)**	**0.04**
HMDB0000247 **(Sorbitol)**	63.00 (57.70)	156.84 (196.34)	110.39 (144.20)	**0.005 (W)**	**0.005**	0.17 (W)	0.11 (W)	0.28 (W)	0.28
HMDB0000251 (**Taurine)**	675.39 (1577.54)	671.00 (1286.50)	449.12 (420.25)	0.69 (W)	0.69	0.99 (W)	0.76	0.86 (W)	0.86
HMDB0000254 ŧ (**Succinic acid)**	50.47 (49.86)	82.25 (73.00)	87.64 (79.98)	**0.001 (W)**	**0.001**	**0.03 (W)**	**0.011**	0.38 (W)	0.37
HMDB0000258 **(Sucrose)**	20.27 (21.89)	37.18 (50.32)	21.62 (17.76)	**0.01 (W)**	**0.01**	0.47 (W)	0.76 (W)	**0.004 (W)**	**0.004**
HMDB0000262 (**Thymine)**	196.47 (192.25)	280.89 (311.95)	272.47 (277.58)	0.19 (W)	0.19	0.18 (W)	0.40 (W)	0.78 (W)	0.78
HMDB0000267 **(Pyroglutamic acid)**	142.27 (131.93)	220.45 (288.42)	154.88 (95.17)	**0.002**	**0.002**	0.20 (W)	0.45	**0.01**	**0.01**
HMDB0000271 (**Sarcosine)**	27.79 (39.82)	31.47 (38.57)	25.23 (17.59)	0.56	0.56	0.36 (W)	0.88	0.64	0.64
HMDB0000294 **(Urea)**	27671.50 (17850.52)	27266.46 (22852.77)	29697.00 (18747.74)	**0.004 (W)**	**0.004**	0.70 (W)	0.16 (W)	0.09 (W)	0.09
HMDB0000296 (**Uridine)**	12.52 (10.12)	24.88 (44.59)	12.58 (11.31)	0.08 (W)	0.08	0.70 (W)	0.45 (W)	**0.005 (W)**	**0.01**
HMDB0000300 ŧ (**Uracil)**	95.61 (91.38)	61.57 (67.85)	68.81 (50.59)	**0.002**	**0.002**	**0.62 (W)**	**0.01**	0.34	0.34
HMDB0000301 (**Urocanic acid)**	14.45 (19.22)	12.85 (12.30)	10.46 (7.72)	0.35	0.35	0.75 (W)	0.34	0.07	0.07
HMDB0000306 (**Tyramine)**	96.02 (106.28)	89.93 (87.73)	94.21 (81.90)	0.41	0.41	0.85 (W)	0.35	0.82	0.82
HMDB0000357 (**3-Hydroxybutyric acid)**	146.83 (269.72)	110.77 (184.82)	91.21 (135.05)	0.08	0.082	0.78 (W)	0.08 (W)	0.79 (W)	0.79
HMDB0000407 (**2-Hydroxy-3-methylbutyric acid)**	14.99 (19.76)	15.72 (18.70)	11.98 (9.72)	0.93 (W)	0.93	0.96 (W)	0.14 (W)	**0.05**	**0.05**

HMDB0000448 **(Adipic acid)**	26.67 (28.86)	38.16 (43.08)	29.13 (30.95)	**0.01**	**0.01**	0.68 (W)	0.90	**0.01**	**0.01**
HMDB0000479 **(Methylhistidine)**	426.48 (1382.88)	181.42 (422.78)	340.32 (1183.63)	0.15 (W)	0.15	0.51 (W)	0.54 (W)	**0.04 (W)**	**0.04**
HMDB0000510 **(Aminoadipic acid)**	56.38 (54.90)	53.21 (49.30)	49.94 (37.81)	0.98 (W)	0.98	0.83 (W)	0.22 (W)	0.33 (W)	0.33
HMDB0000517 **(L-Arginine)**	307.11 (262.96)	351.16 (329.27)	325.38 (208.15)	0.49	0.49	0.64 (W)	0.63	0.25	0.25
HMDB0000538 **(Adenosine triphosphate)**	24.17 (18.08)	35.51 (41.76)	23.44 (18.05)	0.11	0.11	0.86 (W)	0.16	**0.01**	**0.01**
HMDB0000562 **(Creatinine)**	8919.68 (8681.50)	9502.03 (10047.25)	8023.13 (6254.62)	0.18	0.18	0.90 (W)	0.05 (W)	0.63 (W)	0.63
HMDB0000574 **(L-Cysteine)**	210.40 (335.16)	751.82 (1687.31)	356.82 (613.80)	0.09 (W)	0.09	0.33 (W)	0.51 (W)	0.20 (W)	0.20
HMDB0000625 **(Gluconic acid)**	116.10 (135.95)	240.33 (393.28)	113.28 (156.33)	0.36 (W)	0.36	0.72 (W)	0.43 (W)	0.08 (W)	0.08
HMDB0000630 **(Cytosine)**	17.12 (23.26)	17.51 (26.96)	31.07 (101.34)	0.59	0.59	0.51 (W)	0.70 (W)	0.61 (W)	0.61
HMDB0000641 **(Glutamine)**	485.68 (454.62)	672.29 (872.88)	446.40 (431.20)	0.69 (W)	0.69	**0.44 (W)**	**0.01**	**0.02 (W)**	**0.02**
HMDB0000671 **(Indolelactic acid)**	49.43 (40.73)	62.83 (64.40)	57.21 (47.76)	0.21 (W)	0.21	0.70 (W)	0.82 (W)	0.14 (W)	0.14
HMDB0000682 **(Indoxyl sulfate)**	204.72 (154.18)	237.23 (284.00)	270.21 (287.87)	0.64	0.64	0.53 (W)	0.49	0.24	0.24
HMDB0000684 **(Kynurenine)**	44.64 (51.88)	35.19 (31.05)	30.15 (18.58)	0.95	0.950	0.78 (W)	0.09	0.07	0.07
HMDB0000687 **(Leucine)**	22.30 (30.88)	23.80 (25.62)	21.05 (15.33)	0.39	0.39	0.50 (W)	0.72	0.22	0.22
HMDB0000691 **(Malonic acid)**	44.19 (58.93)	78.63 (160.26)	39.99 (48.11)	0.97 (W)	0.97	0.81 (W)	0.40 (W)	0.43 (W)	0.43
HMDB0000696 **(Methionine)**	38.42 (77.50)	58.08 (111.66)	38.51 (71.92)	0.14 (W)	0.14	0.32 (W)	0.61(W)	0.48	0.48
HMDB0000699 **(1-Methylnicotinamide)**	51.37 (50.76)	46.02 (42.02)	60.09 (57.18)	0.79	0.79	0.35 (W)	0.43	0.34	0.34
HMDB0000703 **(Mandelic acid)**	41.04 (38.72)	68.40 (110.46)	180.65 (759.01)	0.34 (W)	0.34	0.59 (W)	0.57(W)	0.70 (W)	0.70

HMDB0000714 **(Hippuric acid)**	587.68 (442.44)	1305.45 (1755.61)	980.52 (845.37)	**0.01 (W)**	**0.01**	0.10 (W)	0.12 (W)	0.56	0.56
HMDB0000718 **(Isovaleric acid)**	15.62 (16.96)	19.34 (24.72)	14.68 (12.66)	0.89 (W)	0.89	0.91 (W)	0.50(W)	0.21 (W)	0.21
HMDB0000754 **(3-Hydroxyisovaleric acid)**	53.77 (62.13)	54.07 (51.00)	51.32 (41.26)	0.87	0.87	0.70 (W)	0.64	0.74	0.74
HMDB0000754.1	44.77 (53.90)	44.76 (43.27)	42.88 (35.42)	0.95 (W)	0.95	0.68 (W)	0.91	0.93 (W)	0.93
HMDB0000765 **(Mannitol)**	165.60 (184.14)	229.87 (323.32)	120.55 (139.37)	0.10 (W)	0.10	0.52 (W)	0.08 (W)	**0.001 (W)**	**0.001**
HMDB0000812 **(N-Acetyl-L-aspartic acid)**	33.94 (33.17)	36.37 (37.50)	34.26 (25.91)	0.56	0.56	0.65 (W)	0.99 (W)	0.73 (W)	0.73
HMDB0000870ⱡ **(Histamine)**	262.46 (402.60)	397.61 (496.59)	608.75 (759.52)	**0.04 (W)**	**0.04**	**0.01 (W)**	**0.004 (W)**	0.33 (W)	0.33
HMDB0000875 **(Trigonelline)**	103.62 (155.43)	219.27 (380.83)	152.43 (183.30)	**0.03**	**0.02**	0.35 (W)	0.35	0.18	0.18
HMDB0000883 **(L-Valine)**	28.48 (24.05)	31.65 (31.03)	30.47 (20.97)	0.87	0.87	0.67 (W)	0.48	0.36	0.36
HMDB0000902 **(NAD)**	8.28 (9.34)	5.01 (4.00)	6.98 (6.26)	0.20	0.20	0.85 (W)	0.28	0.77	0.77
HMDB0000906 **(Trimethylamine)**	8.65 (8.95)	13.56 (17.33)	8.86 (7.52)	0.06 (W)	0.06	0.77 (W)	0.76 (W)	**0.01**	**0.01**
HMDB0000925 **(Trimethylamine N-oxide)**	422.10 (501.47)	375.42 (554.29)	549.05 (1406.16)	0.23	0.23	0.82 (W)	0.39 (W)	0.56 (W)	0.56
HMDB0000929 **(L-Tryptophan)**	160.87 (141.47)	188.33 (203.50)	161.69 (133.93)	0.55	0.55	0.92 (W)	0.89(W)	0.57 (W)	0.57
HMDB0000958 **(trans-Aconitic acid)**	31.44 (31.89)	56.75 (73.71)	47.43 (76.62)	**0.02 (W)**	**0.02**	0.51 (W)	0.88 (W)	**0.04 (W)**	**0.04**
HMDB0001341 **(ADP)**	14.68 (13.70)	25.20 (32.70)	16.12 (12.64)	**0.01 (W)**	**0.01**	0.59 (W)	0.74(W)	**0.03**	**0.03**
HMDB0001522 **(Methylguanidine)**	70.49 (116.97)	65.79 (95.04)	49.26 (41.22)	0.63 (W)	0.63	0.92 (W)	0.20 (W)	0.30	0.30
HMDB0001525 **(Imidazole)**	216.85 (289.41)	297.79 (422.00)	334.97 (488.48)	0.16 (W)	0.16	0.25 (W)	0.38(W)	0.72	0.72
HMDB0001565 **(Phosphorylcholine)**	30.30 (48.42)	40.45 (52.46)	31.52 (35.52)	0.47	0.47	0.40 (W)	0.40	0.99	0.99

HMDB0001659 (**Acetone**)	19.50 (20.99)	21.56 (17.70)	18.22 (11.07)	0.36	0.36	0.22 (W)	0.91 (W)	0.26 (W)	0.26
HMDB0001844 (**Methylsuccinic acid**)	13.30 (10.62)	14.49 (13.71)	10.66 (7.88)	0.79 (W)	0.79	0.44 (W)	0.10 (W)	0.08	0.08
HMDB0001847 (**Caffeine**)	23.74 (21.69)	25.94 (29.59)	30.43 (26.95)	0.96	0.96	0.48 (W)	0.40(W)	0.65 (W)	0.65
HMDB0001858 (**p-Cresol**)	107.99 (92.31)	120.82 (139.46)	126.80 (128.24)	0.65	0.65	0.74 (W)	0.86	0.55	0.55
HMDB0001863 (**2-Hydroxyvaleric acid**)	28.99 (37.63)	23.89 (38.23)	17.32 (16.78)	0.36	0.36	**0.46 (W)**	**0.02**	0.13	0.13
HMDB0001873 (**Isobutyric acid**)	34.93 (34.23)	49.79 (73.92)	40.07 (36.28)	0.99 (W)	0.99	0.53 (W)	0.94	0.59 (W)	0.59
HMDB0001873.1	36.40 (34.53)	53.76 (89.25)	47.95 (69.59)	0.88 (W)	0.88	0.72 (W)	0.46 (W)	0.56 (W)	0.56
HMDB0001875 **(Methanol)**	236.09 (271.44)	304.77 (344.52)	1127.76 (3537.81)	**0.05 (W)**	**0.05**	0.03 (W)	0.05 (W)	0.91 (W)	0.91
HMDB0001881 (**Propylene glycol**)	5.46 (10.74)	5.27 (5.97)	5.61 (8.37)	0.27	0.27	0.36 (W)	0.69 (W)	0.51 (W)	0.51
HMDB0002322 (**Cadaverine**)	27.05 (29.14)	25.48 (24.20)	27.63 (21.43)	0.95	0.95	0.47 (W)	0.79	0.68	0.68
HMDB0003355 **(5-Aminopentanoic acid)**	23.09 (39.38)	57.89 (89.83)	32.06 (37.09)	**0.01 (W)**	**0.01**	0.14 (W)	0.32 (W)	0.09 (W)	0.09
HMDB0003911 (**3-Aminoisobutanoic acid**)	171.94 (284.37)	189.42 (339.50)	139.21 (178.99)	0.91 (W)	0.91	0.66 (W)	0.97(W)	0.98	0.98
HMDB0004983 (**Dimethyl sulfone**)	50.73 (40.36)	45.35 (34.32)	55.94 (35.26)	0.94	0.94	0.41 (W)	0.56	0.56	0.56
HMDB0011635 (**p-Cresol sulfate**)	30.41 (35.04)	36.75 (51.79)	30.44 (34.22)	0.21	0.21	0.42 (W)	0.63	0.38	0.38
HMDB0031645 (**Acetamide**)	24.04 (22.50)	33.34 (35.03)	42.64 (38.48)	0.27	0.27	**0.06 (W)**	**0.01**	0.28	0.28
HMDB0059655[ɫ] (**2-Hydroxyglutarate**)	254.93 (272.00)	562.82 (855.46)	379.36 (336.66)	**0.01**	**0.01**	**0.10 (W)**	**0.005**	0.82	0.82

[ɫ] Metabolites that were consistently significantly different from baseline at both 2 and 10-weeks.

p-value with (W) is calculated by the Wilcoxon Mann Whitney test

Supplementary Table S2: Results of enrichment analysis when urine metabolomics data for baseline and week 2 compared

	Total Cmpd	Hits	Statistic Q	Raw p	Holm p	FDR
Citric Acid Cycle	32	10	7.93	3.10E-06	0.0003	0.0003
Warburg Effect	58	13	7.19	5.71E-06	0.0005	0.0003
Amino Sugar Metabolism	33	6	8.07	3.37E-05	0.003	0.0007
Galactose Metabolism	38	9	7.62	4.19E-05	0.004	0.0007
Arginine and Proline Metabolism	53	15	5.95	5.38E-05	0.005	0.0007
Mitochondrial Electron Transport Chain	19	5	9.30	5.49E-05	0.005	0.0007
Pyruvate Metabolism	48	9	6.22	5.68E-05	0.005	0.0007
Cysteine Metabolism	26	8	6.49	5.99E-05	0.005	0.0007
Glutamate Metabolism	49	14	5.09	7.16E-05	0.006	0.0007
Alanine Metabolism	17	9	6.63	7.56E-05	0.007	0.0007
Urea Cycle	29	13	6.20	0.0002	0.01	0.001
Transfer of Acetyl Groups into Mitochondria	22	7	6.69	0.0002	0.01	0.001
Purine Metabolism	74	14	5.86	0.0003	0.03	0.002
Glutathione Metabolism	21	7	6.24	0.0004	0.03	0.002
Aspartate Metabolism	35	12	5.45	0.0004	0.03	0.002
Propanoate Metabolism	42	9	5.42	0.0004	0.03	0.002
Phenylalanine and Tyrosine Metabolism	28	8	8.04	0.0004	0.03	0.002
Ethanol Degradation	19	5	6.62	0.0005	0.039	0.003
Histidine Metabolism	43	10	5.30	0.0005	0.042	0.003
Nicotinate and Nicotinamide Metabolism	37	7	6.37	0.0006	0.05	0.003
Pyrimidine Metabolism	59	7	5.68	0.0007	0.05	0.003
Starch and Sucrose Metabolism	31	5	6.83	0.0007	0.05	0.003
Glycine and Serine Metabolism	59	19	4.03	0.0007	0.05	0.003

Pantothenate and CoA Biosynthesis	21	4	7.76	0.0007	0.05	0.003
Pentose Phosphate Pathway	29	3	9.52	0.0008	0.06	0.003
Riboflavin Metabolism	20	3	9.52	0.0008	0.06	0.003
Thiamine Metabolism	9	3	9.52	0.0008	0.06	0.003
Tyrosine Metabolism	72	9	6.47	0.0008	0.06	0.003
Nucleotide Sugars Metabolism	20	4	7.53	0.0009	0.06	0.003
Butyrate Metabolism	19	5	6.02	0.001	0.07	0.003
Lactose Degradation	9	4	8.41	0.001	0.07	0.003
Folate Metabolism	29	5	6.52	0.001	0.08	0.004
Phytanic Acid Peroxisomal Oxidation	26	5	5.97	0.002	0.10	0.005
Selenoamino Acid Metabolism	28	7	5.27	0.002	0.12	0.005
Methionine Metabolism	43	11	4.03	0.002	0.13	0.006
Glycolysis	25	5	5.94	0.002	0.14	0.006
Sphingolipid Metabolism	40	6	5.74	0.002	0.14	0.006
Oxidation of Branched Chain Fatty Acids	26	6	5.38	0.002	0.14	0.006
Ammonia Recycling	32	14	3.88	0.003	0.15	0.006
Gluconeogenesis	35	7	4.93	0.003	0.17	0.007
Phenylacetate Metabolism	9	4	6.38	0.003	0.18	0.007
Betaine Metabolism	21	6	4.72	0.004	0.19	0.008
Mitochondrial Beta-Oxidation of Medium Chain Saturated Fatty Acids	27	3	6.43	0.004	0.20	0.008
Phosphatidylcholine Biosynthesis	14	5	5.21	0.004	0.21	0.009
Arachidonic Acid Metabolism	69	1	11.63	0.004	0.23	0.009
Glycerolipid Metabolism	25	4	5.69	0.005	0.23	0.009
Valine, Leucine and Isoleucine Degradation	60	11	4.12	0.005	0.24	0.01
Fructose and Mannose Degradation	32	5	5.23	0.005	0.24	0.01

Inositol Phosphate Metabolism	26	4	5.62	0.005	0.24	0.01
Phosphatidylinositol Phosphate Metabolism	17	3	6.67	0.005	0.24	0.01
Lactose Synthesis	20	3	7.2	0.006	0.25	0.01
Catecholamine Biosynthesis	20	1	11.01	0.006	0.25	0.01
Thyroid hormone synthesis	13	1	11.01	0.006	0.25	0.01
Phosphatidylethanolamine Biosynthesis	12	5	5.05	0.006	0.25	0.01
Fatty acid Metabolism	43	4	5.18	0.006	0.25	0.01
Mitochondrial Beta-Oxidation of Short Chain Saturated Fatty Acids	27	4	5.18	0.006	0.25	0.01
Mitochondrial Beta-Oxidation of Long Chain Saturated Fatty Acids	28	4	5.18	0.007	0.25	0.01
Beta-Alanine Metabolism	34	8	3.99	0.006	0.25	0.01
Glucose-Alanine Cycle	13	6	4.50	0.0067	0.25	0.01
Lysine Degradation	30	5	4.66	0.007	0.25	0.01
Threonine and 2-Oxobutanoate Degradation	20	4	5.38	0.008	0.28	0.01
D-Arginine and D-Ornithine Metabolism	11	1	10.10	0.008	0.28	0.01
Sulfate/Sulfite Metabolism	22	2	7.82	0.008	0.28	0.01
Trehalose Degradation	11	2	7.82	0.008	0.28	0.01
Steroid Biosynthesis	48	3	6.16	0.009	0.29	0.014
Inositol Metabolism	33	5	4.58	0.01	0.29	0.01
Beta Oxidation of Very Long Chain Fatty Acids	17	4	5.14	0.01	0.37	0.02
Fatty Acid Biosynthesis	35	4	4.61	0.01	0.38	0.02
Carnitine Synthesis	22	6	3.63	0.02	0.61	0.03
Malate-Aspartate Shuttle	10	4	3.93	0.03	0.77	0.04
Bile Acid Biosynthesis	65	5	3.63	0.03	0.81	0.04

Biotin Metabolism	8	2	4.99	0.04	0.85	0.05
Ketone Body Metabolism	13	4	3.63	0.04	0.9899	0.06
Spermidine and Spermine Biosynthesis	18	2	4.49	0.05	1	0.06
Pyruvaldehyde Degradation	10	1	5.61	0.05	1	0.07
Tryptophan Metabolism	60	8	2.84	0.06	1	0.08
Phospholipid Biosynthesis	29	4	3.22	0.07	1	0.09
Vitamin B6 Metabolism	20	1	3.36	0.14	1	0.16
Pterine Biosynthesis	29	2	2.66	0.17	1	0.20
Androgen and Estrogen Metabolism	33	2	2.66	0.17	1	0.20
Androstenedione Metabolism	24	2	2.66	0.17	1	0.20
Vitamin K Metabolism	14	2	2.46	0.19	1	0.21
Fatty Acid Elongation In Mitochondria	35	1	2.47	0.20	1	0.21
Retinol Metabolism	37	1	2.47	0.20	1	0.21
Glycerol Phosphate Shuttle	11	1	2.47	0.20	1	0.21
Steroidogenesis	43	1	2.47	0.20	1	0.21
Plasmalogen Synthesis	26	1	2.47	0.20	1	0.21
De Novo Triacylglycerol Biosynthesis	9	1	2.47	0.20	1	0.21
Cardiolipin Biosynthesis	11	1	2.47	0.20	1	0.21
Estrone Metabolism	24	1	2.47	0.20	1	0.21
Methylhistidine Metabolism	4	2	1.48	0.38	1	0.39
Homocysteine Degradation	9	2	1.25	0.44	1	0.44
Porphyrin Metabolism	40	2	1.25	0.44	1	0.44
Taurine and Hypotaurine Metabolism	12	2	1.25	0.44	1	0.44
Caffeine Metabolism	24	2	1.23	0.44	1	0.44

Supplementary Table S3: Fruit and vegetable intake correlation with significant metabolites at each timepoint

Metabolite	Baseline		Week 2		Week 10	
	Fruit	Vegetable	Fruit	Vegetable	Fruit	Vegetable
Acetic acid	-0.10	-0.18	-0.08	-0.15	0.02	-0.01
Dimethylamine	-0.17	-0.14	0.11	-0.01	0.06	-0.17
Choline	-0.12	-0.46	0.06	-0.19	0.13	-0.12
Fumaric acid	-0.07	-0.13	0.12	-0.07	0.28	-0.05
Glutamic acid	-0.23	-0.36	0.05	-0.14	0.31	-0.22
L-tyrosine	0.01	-0.25	0.26	0.01	-0.31	-0.13
Histidine	-0.13	-0.22	0.06	-0.05	0.17	-0.24
Succinic acid	-0.07	-0.24	0.15	0.18	0.10	-0.03
Uracil	-0.12	-0.24	0.11	-0.01	-0.06	-0.17
Histamine	0.11	-0.06	0.04	-0.12	0.27	-0.13
2-hydroxyglutarate	-0.04	-0.21	0.24	-0.02	0.17	-0.20

Spearman correlations (r_s) < 0.2 were classified as weak, 0.2–0.6 moderate and > 0.6 strong
All p-values >0.05

Supplementary Table S4: Results of enrichment analysis when urine metabolomics data for baseline and week 10 compared

	Total Cmpd	Hits	Statistic Q	Raw p	Holm p	FDR
Starch and Sucrose Metabolism	31	5	9.24	1.28E-05	0.001	0.001
Inositol Metabolism	33	5	9.15	2.36E-05	0.002	0.001
Warburg Effect	58	13	5.81	7.30E-05	0.007	0.002
Amino Sugar Metabolism	33	6	7.48	7.80E-05	0.007	0.002
Aspartate Metabolism	35	12	5.76	0.0002	0.02	0.004
Ammonia Recycling	32	14	4.90	0.0003	0.03	0.005
Histidine Metabolism	43	10	5.15	0.0007	0.06	0.008
Citric Acid Cycle	32	10	5.10	0.0007	0.06	0.008
Purine Metabolism	74	14	5.36	0.0007	0.06	0.008
Phenylalanine and Tyrosine Metabolism	28	8	7.26	0.001	0.10	0.01
Glutamate Metabolism	49	14	4.22	0.002	0.13	0.01
Mitochondrial Electron Transport Chain	19	5	6.00	0.002	0.14	0.01
Urea Cycle	29	13	4.87	0.002	0.14	0.01
Beta-Alanine Metabolism	34	8	5.00	0.002	0.15	0.01
Glucose-Alanine Cycle	13	6	5.26	0.003	0.23	0.02
Lysine Degradation	30	5	5.64	0.003	0.24	0.02
Arginine and Proline Metabolism	53	15	3.95	0.004	0.28	0.02
Arachidonic Acid Metabolism	69	1	12.40	0.004	0.29	0.02
Cysteine Metabolism	26	8	4.52	0.004	0.29	0.02
Pyruvaldehyde Degradation	10	1	12.03	0.004	0.33	0.02
Tyrosine Metabolism	72	9	5.24	0.005	0.34	0.02
Nicotinate and Nicotinamide Metabolism	37	7	4.59	0.005	0.41	0.02
Alanine Metabolism	17	9	4.02	0.01	0.61	0.03

Pyruvate Metabolism	48	9	3.84	0.01	0.77	0.04
Catecholamine Biosynthesis	20	1	9.55	0.01	0.82	0.04
Thyroid hormone synthesis	13	1	9.55	0.01	0.82	0.04
Glycerolipid Metabolism	25	4	4.82	0.01	0.92	0.05
Pyrimidine Metabolism	59	7	4.06	0.01	1	0.05
Carnitine Synthesis	22	6	4.00	0.02	1	0.05
Tryptophan Metabolism	60	8	3.66	0.02	1	0.05
Folate Metabolism	29	5	4.33	0.02	1	0.05
Propanoate Metabolism	42	9	3.54	0.02	1	0.06
Phenylacetate Metabolism	9	4	4.16	0.03	1	0.08
Gluconeogenesis	35	7	3.63	0.03	1	0.08
Glycolysis	25	5	3.95	0.03	1	0.08
Malate-Aspartate Shuttle	10	4	4.09	0.03	1	0.08
Butyrate Metabolism	19	5	3.73	0.03	1	0.09
Valine, Leucine and Isoleucine Degradation	60	11	3.23	0.03	1	0.09
Transfer of Acetyl Groups into Mitochondria	22	7	3.34	0.04	1	0.10
Biotin Metabolism	8	2	4.75	0.04	1	0.11
Glycine and Serine Metabolism	59	19	2.63	0.05	1	0.11
Phytanic Acid Peroxisomal Oxidation	26	5	3.43	0.0	1	0.11
Galactose Metabolism	38	9	3.05	0.05	1	0.11
Ethanol Degradation	19	5	3.29	0.06	1	0.12
Fatty Acid Biosynthesis	35	4	3.49	0.06	1	0.12
Betaine Metabolism	21	6	2.96	0.07	1	0.15
Oxidation of Branched Chain Fatty Acids	26	6	2.96	0.08	1	0.16
Vitamin K Metabolism	14	2	3.79	0.08	1	0.17
Glutathione Metabolism	21	7	2.83	0.09	1	0.17

Ketone Body Metabolism	13	4	2.97	0.10	1	0.19
Methylhistidine Metabolism	4	2	3.50	0.10	1	0.19
Vitamin B6 Metabolism	20	1	3.80	0.12	1	0.21
Fatty acid Metabolism	43	4	2.75	0.13	1	0.22
Mitochondrial Beta-Oxidation of Short Chain Saturated Fatty Acids	27	4	2.75	0.13	1	0.22
Mitochondrial Beta-Oxidation of Long Chain Saturated Fatty Acids	28	4	2.75	0.13	1	0.22
Mitochondrial Beta-Oxidation of Medium Chain Saturated Fatty Acids	27	3	2.87	0.13	1	0.23
Methionine Metabolism	43	11	2.27	0.14	1	0.23
Selenoamino Acid Metabolism	28	7	2.44	0.14	1	0.23
Lactose Degradation	9	4	2.58	0.16	1	0.24
Pterine Biosynthesis	29	2	2.85	0.16	1	0.24
Androgen and Estrogen Metabolism	33	2	2.85	0.16	1	0.24
Androstenedione Metabolism	24	2	2.85	0.16	1	0.24
Spermidine and Spermine Biosynthesis	18	2	2.85	0.16	1	0.24
Nucleotide Sugars Metabolism	20	4	2.44	0.18	1	0.26
Steroid Biosynthesis	48	3	2.47	0.18	1	0.27
Pentose Phosphate Pathway	29	3	2.40	0.20	1	0.28
Riboflavin Metabolism	20	3	2.40	0.20	1	0.28
Thiamine Metabolism	9	3	2.40	0.20	1	0.28
Sphingolipid Metabolism	40	6	2.12	0.22	1	0.30
D-Arginine and D-Ornithine Metabolism	11	1	2.04	0.25	1	0.32
Phosphatidylethanolamine Biosynthesis	12	5	1.98	0.27	1	0.32
Phospholipid Biosynthesis	29	4	1.99	0.27	1	0.32

Lactose Synthesis	20	3	1.97	0.27	1	0.32
Phosphatidylcholine Biosynthesis	14	5	1.94	0.28	1	0.32
Fatty Acid Elongation in Mitochondria	35	1	1.81	0.28	1	0.32
Retinol Metabolism	37	1	1.81	0.28	1	0.32
Glycerol Phosphate Shuttle	11	1	1.81	0.28	1	0.32
Steroidogenesis	43	1	1.81	0.28	1	0.32
Plasmalogen Synthesis	26	1	1.81	0.28	1	0.32
De Novo Triacylglycerol Biosynthesis	9	1	1.81	0.28	1	0.32
Cardiolipin Biosynthesis	11	1	1.81	0.28	1	0.32
Estrone Metabolism	24	1	1.81	0.28	1	0.32
Pantothenate and CoA Biosynthesis	21	4	1.94	0.28	1	0.32
Sulfate/Sulfite Metabolism	22	2	1.76	0.31	1	0.35
Trehalose Degradation	11	2	1.76	0.31	1	0.35
Porphyrin Metabolism	40	2	1.70	0.33	1	0.37
Beta Oxidation of Very Long Chain Fatty Acids	17	4	1.49	0.42	1	0.45
Threonine and 2-Oxobutanoate Degradation	20	4	1.44	0.44	1	0.47
Bile Acid Biosynthesis	65	5	1.41	0.47	1	0.50
Inositol Phosphate Metabolism	26	4	1.34	0.48	1	0.51
Phosphatidylinositol Phosphate Metabolism	17	3	1.19	0.50	1	0.53
Fructose and Mannose Degradation	32	5	1.30	0.51	1	0.53
Homocysteine Degradation	9	2	0.94	0.55	1	0.56
Caffeine Metabolism	24	2	0.91	0.56	1	0.56
Taurine and Hypotaurine Metabolism	12	2	0.36	0.80	1	0.780

Supplementary Table S5: Results of enrichment analysis when urine metabolomics data for Week 2 and Week 10 compared

	Total Cmpd	Hits	Statistic Q	Raw p	Holm p	FDR
Starch and Sucrose Metabolism	31	5	9.76	0.0002	0.02	0.01
Pyrimidine Metabolism	59	7	6.68	0.0004	0.03	0.01
Phenylacetate Metabolism	9	4	8.96	0.0004	0.03	0.01
Inositol Metabolism	33	5	8.19	0.0005	0.05	0.01
Amino Sugar Metabolism	33	6	6.47	0.0008	0.07	0.02
Galactose Metabolism	38	9	6.69	0.001	0.10	0.02
Transfer of Acetyl Groups into Mitochondria	22	7	5.99	0.001	0.11	0.02
Lactose Synthesis	20	3	10.23	0.001	0.12	0.02
Glycolysis	25	5	6.82	0.002	0.16	0.02
Warburg Effect	58	13	4.71	0.002	0.19	0.02
Lactose Degradation	9	4	7.85	0.002	0.21	0.02
Glutamate Metabolism	49	14	4.00	0.003	0.25	0.02
Sphingolipid Metabolism	40	6	5.32	0.005	0.43	0.03
Nicotinate and Nicotinamide Metabolism	37	7	5.14	0.006	0.46	0.03
Glycerolipid Metabolism	25	4	6.40	0.006	0.49	0.03
Alanine Metabolism	17	9	4.43	0.006	0.50	0.03
Histidine Metabolism	43	10	4.27	0.006	0.50	0.03
Gluconeogenesis	35	7	4.92	0.007	0.53	0.03
Phosphatidylinositol Phosphate Metabolism	17	3	7.29	0.007	0.54	0.03
Ammonia Recycling	32	14	3.69	0.01	0.69	0.04
Sulfate/Sulfite Metabolism	22	2	8.44	0.01	0.81	0.05
Trehalose Degradation	11	2	8.44	0.01	0.81	0.05
Aspartate Metabolism	35	12	3.67	0.01	0.88	0.05
Urea Cycle	29	13	3.69	0.01	0.88	0.05

Inositol Phosphate Metabolism	26	4	5.47	0.01	0.88	0.05
Pentose Phosphate Pathway	29	3	6.83	0.01	0.96	0.05
Riboflavin Metabolism	20	3	6.83	0.01	0.96	0.05
Thiamine Metabolism	9	3	6.83	0.01	0.96	0.05
Glutathione Metabolism	21	7	4.10	0.02	1	0.06
Pantothenate and CoA Biosynthesis	21	4	5.25	0.02	1	0.06
Steroid Biosynthesis	48	3	5.63	0.02	1	0.07
Beta Oxidation of Very Long Chain Fatty Acids	17	4	4.90	0.03	1	0.07
Purine Metabolism	74	14	3.13	0.03	1	0.07
Threonine and 2-Oxobutanoate Degradation	20	4	4.57	0.03	1	0.07
Spermidine and Spermine Biosynthesis	18	2	5.37	0.03	1	0.08
Mitochondrial Electron Transport Chain	19	5	4.23	0.03	1	0.08
Pyruvate Metabolism	48	9	3.42	0.03	1	0.08
Nucleotide Sugars Metabolism	20	4	4.40	0.03	1	0.08
Mitochondrial Beta-Oxidation of Medium Chain Saturated Fatty Acids	27	3	4.60	0.03	1	0.08
Citric Acid Cycle	32	10	3.29	0.03	1	0.08
Fructose and Mannose Degradation	32	5	4.00	0.04	1	0.08
Biotin Metabolism	8	2	5.08	0.04	1	0.08
Phosphatidylcholine Biosynthesis	14	5	3.84	0.04	1	0.08
Phosphatidylethanolamine Biosynthesis	12	5	3.94	0.04	1	0.09
Phytanic Acid Peroxisomal Oxidation	26	5	3.80	0.04	1	0.09
Glycine and Serine Metabolism	59	19	2.70	0.04	1	0.09
Oxidation of Branched Chain Fatty Acids	26	6	3.61	0.04	1	0.09
Ethanol Degradation	19	5	3.56	0.05	1	0.09
Bile Acid Biosynthesis	65	5	3.60	0.05	1	0.09
Cysteine Metabolism	26	8	3.10	0.06	1	0.11

Selenoamino Acid Metabolism	28	7	3.20	0.06	1	0.11
Glucose-Alanine Cycle	13	6	3.13	0.06	1	0.11
Fatty acid Metabolism	43	4	3.46	0.06	1	0.11
Mitochondrial Beta-Oxidation of Short Chain Saturated Fatty Acids	27	4	3.46	0.06	1	0.11
Mitochondrial Beta-Oxidation of Long Chain Saturated Fatty Acids	28	4	3.46	0.06	1	0.11
Folate Metabolism	29	5	3.39	0.07	1	0.11
Arginine and Proline Metabolism	53	15	2.57	0.08	1	0.13
Butyrate Metabolism	19	5	3.12	0.08	1	0.13
Propanoate Metabolism	42	9	2.80	0.08	1	0.13
Valine, Leucine and Isoleucine Degradation	60	11	2.64	0.09	1	0.14
Methylhistidine Metabolism	4	2	3.71	0.09	1	0.14
Phenylalanine and Tyrosine Metabolism	28	8	2.59	0.12	1	0.19
Pyruvaldehyde Degradation	10	1	3.39	0.14	1	0.21
D-Arginine and D-Ornithine Metabolism	11	1	3.31	0.15	1	0.22
Tryptophan Metabolism	60	8	2.37	0.15	1	0.22
Beta-Alanine Metabolism	34	8	2.24	0.18	1	0.26
Betaine Metabolism	21	6	2.03	0.25	1	0.36
Vitamin K Metabolism	14	2	1.73	0.34	1	0.47
Methionine Metabolism	43	11	1.63	0.40	1	0.56
Fatty Acid Biosynthesis	35	4	1.25	0.52	1	0.71
Ketone Body Metabolism	13	4	0.92	0.67	1	0.90
Homocysteine Degradation	9	2	0.51	0.73	1	0.96
Porphyrin Metabolism	40	2	0.48	0.74	1	0.96
Tyrosine Metabolism	72	9	0.90	0.78	1	0.98
Taurine and Hypotaurine Metabolism	12	2	0.34	0.81	1	0.98
Phospholipid Biosynthesis	29	4	0.59	0.83	1	0.98
Catecholamine Biosynthesis	20	1	0.07	0.84	1	0.98

Thyroid hormone synthesis	13	1	0.07	0.84	1	0.98
Arachidonic Acid Metabolism	69	1	0.03	0.89	1	0.98
Fatty Acid Elongation In Mitochondria	35	1	0.02	0.90	1	0.98
Retinol Metabolism	37	1	0.02	0.90	1	0.98
Glycerol Phosphate Shuttle	11	1	0.02	0.90	1	0.98
Steroidogenesis	43	1	0.02	0.90	1	0.98
Plasmalogen Synthesis	26	1	0.02	0.90	1	0.98
De Novo Triacylglycerol Biosynthesis	9	1	0.02	0.90	1	0.98
Cardiolipin Biosynthesis	11	1	0.02	0.90	1	0.98
Estrone Metabolism	24	1	0.02	0.90	1	0.98
Carnitine Synthesis	22	6	0.52	0.92	1	0.98
Vitamin B6 Metabolism	20	1	0.02	0.92	1	0.98
Lysine Degradation	30	5	0.31	0.96	1	0.99
Caffeine Metabolism	24	2	0.02	0.99	1	0.99
Pterine Biosynthesis	29	2	0.02	0.99	1	0.99
Androgen and Estrogen Metabolism	33	2	0.02	0.99	1	0.99
Androstenedione Metabolism	24	2	0.02	0.99	1	0.99
Malate-Aspartate Shuttle	10	4	0.11	0.99	1	0.99
	Total Cmpd	Hits	Statistic Q	Raw p	Holm p	FDR

Supplementary Table S6: Baseline – Week 10 Classification using features. Week 10 being the positive class.

Model	Acc	AUC	Recall	Prec	F1	Kappa	MCC
Ridge Classifier	0.87	0.96	0.87	0.90	0.86	0.74	0.76
Linear Discriminant Analysis	0.87	0.96	0.87	0.90	0.86	0.74	0.76
SVM - Linear Kernel	0.85	0.94	0.85	0.89	0.85	0.71	0.74
Logistic Regression	0.83	0.94	0.83	0.87	0.82	0.65	0.69
Extra Trees Classifier	0.83	0.91	0.83	0.84	0.80	0.66	0.70
Naive Bayes	0.77	0.90	0.77	0.81	0.76	0.55	0.58
Quadratic Discriminant Analysis	0.80	0.88	0.80	0.87	0.79	0.61	0.66
Ada Boost Classifier	0.74	0.87	0.74	0.77	0.73	0.49	0.51
Light Gradient Boosting	0.75	0.84	0.75	0.79	0.74	0.50	0.54
K Neighbors Classifier	0.78	0.83	0.78	0.82	0.77	0.57	0.60
Extreme Gradient Boosting	0.78	0.81	0.78	0.83	0.77	0.56	0.60
Random Forest Classifier	0.77	0.81	0.77	0.79	0.75	0.53	0.55
Decision Tree Classifier	0.75	0.75	0.75	0.77	0.74	0.50	0.52
Gradient Boosting Classifier	0.69	0.73	0.69	0.71	0.68	0.39	0.40
Dummy Classifier	0.51	0.50	0.51	0.27	0.35	0.00	0.00

Footnote: Acc, Accuracy; AUC, Area Under the Curve; Prec, Precision; F1, F1-Score; Kappa, Cohen’s Kappa Statistic; MCC, Matthews Correlation Coefficient.

Supplementary Table S7: Features selected for each time point

**Baseline – Week 10 Features**	**Baseline – Week 2 Features**	**Week 2 - Week 10 Features**
Acetone	Citrate	Indoxylsulfate
Anserine	Dimethylamine	Creatine
Dimethylamine	Formate	Guanosine
Histamine	Glucuronate	TrimethylamineNoxide
Pyruvate	Kynurenine	Uridine
Thymine	Pyridoxine	Methylhistidine
Glycerol	Sucrose	Dimethylsulfone
totalSODI	Uracil	totalIRON
totalSFAT	totalARFSVEG	totalNIAC
Average_24hr_fruit_serves	Average_24hr_fruit_serves	Average_24hr_fruit_serves

Supplementary Table S8: Baseline – Week 2 Classification using features. Week 2 being the positive class.

Model	Acc	AUC	Recall	Prec	F1	Kappa	MCC
Ridge Classifier	0.62	0.75	0.62	0.64	0.61	0.24	0.25
Linear Discriminant Analysis	0.62	0.74	0.62	0.64	0.61	0.24	0.25
Logistic Regression	0.63	0.73	0.63	0.65	0.62	0.27	0.28
Ada Boost Classifier	0.63	0.69	0.63	0.65	0.62	0.25	0.27
Naive Bayes	0.60	0.67	0.60	0.59	0.57	0.19	0.21
SVM - Linear Kernel	0.63	0.65	0.63	0.66	0.60	0.25	0.28
Light Gradient Boosting Machine	0.56	0.64	0.56	0.57	0.53	0.13	0.15
Extreme Gradient Boosting	0.56	0.61	0.56	0.57	0.54	0.13	0.13
Random Forest Classifier	0.54	0.61	0.54	0.52	0.49	0.08	0.11
Quadratic Discriminant Analysis	0.59	0.61	0.59	0.60	0.56	0.18	0.21
Extra Trees Classifier	0.51	0.54	0.51	0.50	0.47	0.02	0.01
Gradient Boosting Classifier	0.47	0.54	0.47	0.44	0.43	-0.07	-0.06
K Neighbors Classifier	0.54	0.51	0.54	0.54	0.52	0.08	0.08
Decision Tree Classifier	0.50	0.50	0.50	0.52	0.49	0.00	0.01
Dummy Classifier	0.50	0.50	0.50	0.25	0.33	0.00	0.00

Footnote: Acc, Accuracy; AUC, Area Under the Curve; Prec, Precision; F1, F1-Score; Kappa, Cohen’s Kappa Statistic; MCC, Matthews Correlation Coefficient.

Supplementary Table S9: Week 2 – Week 10 Classification using features. Week 10 being the positive class.

Model	Acc	AUC	Recall	Prec	F1	Kappa	MCC
Ridge Classifier	0.54	0.60	0.54	0.53	0.51	0.08	0.08
Light Gradient Boosting Machine	0.54	0.60	0.54	0.48	0.49	0.07	0.04
Linear Discriminant Analysis	0.54	0.60	0.54	0.53	0.51	0.08	0.08
Quadratic Discriminant Analysis	0.59	0.58	0.59	0.56	0.54	0.18	0.20
Extreme Gradient Boosting	0.55	0.58	0.55	0.52	0.51	0.09	0.09
Logistic Regression	0.53	0.58	0.53	0.51	0.50	0.06	0.05
Extra Trees Classifier	0.51	0.56	0.51	0.47	0.46	0.01	0.00
Decision Tree Classifier	0.56	0.56	0.56	0.52	0.51	0.12	0.11
Naive Bayes	0.51	0.55	0.51	0.46	0.45	0.03	0.04
SVM - Linear Kernel	0.52	0.54	0.52	0.46	0.48	0.05	0.01
Random Forest Classifier	0.56	0.54	0.56	0.54	0.52	0.13	0.13
Gradient Boosting Classifier	0.53	0.50	0.53	0.52	0.50	0.06	0.07
Dummy Classifier	0.49	0.50	0.49	0.24	0.32	0.00	0.00
K Neighbors Classifier	0.54	0.50	0.54	0.53	0.51	0.07	0.09
Ada Boost Classifier	0.50	0.47	0.50	0.44	0.45	-0.01	-0.01

Footnote: Acc, Accuracy; AUC, Area Under the Curve; Prec, Precision; F1, F1-Score; Kappa, Cohen's Kappa Statistic; MCC, Matthews Correlation Coefficient.
